# Continuous intravenous anaesthesia with sufentanil and midazolam in medetomidine premedicated New Zealand White rabbits

**DOI:** 10.1186/1746-6148-9-21

**Published:** 2013-01-28

**Authors:** Patricia Hedenqvist, Anna Edner, Åsa Fahlman, Marianne Jensen-Waern

**Affiliations:** 1 Department of Clinical Sciences, Faculty of Veterinary Medicine and Animal Science, Swedish University of Agricultural Sciences, P.O. Box 7054, SE, 750 07, Uppsala, Sweden

**Keywords:** Opioid, Benzodiazepine, Alpha-2-agonist, TIVA, Rabbit, Sufentanil, Midazolam, Respiratory depression, Hypotension

## Abstract

**Background:**

Anaesthesia in rabbits is associated with a high mortality rate, compared to that in cats and dogs. Total intravenous anaesthesia (TIVA) with drugs that provide cardiovascular stability and are rapidly metabolised could be of benefit for use in rabbits. The aim was to evaluate cardiorespiratory effects of TIVA with sufentanil-midazolam in eight New Zealand White rabbits. Subcutaneous premedication with medetomidine (0.1 mg/kg BW) was followed by IV administration of a mixture of 2.5 μg/mL sufentanil and 0.45 mg/mL midazolam at a rate of 0.3 mL/kg BW/h for anaesthetic induction. Additionally, intravenous boluses of 0.1 mL of the mixture were administered every 20 s until the righting reflex was lost. Following endotracheal intubation, anaesthesia was maintained for 60 min with an infusion rate adjusted to supress the pedal withdrawal reflex. Air and oxygen (1:2) were delivered at 3 L/min. Physiological variables were recorded before induction and at predefined time points during and after anaesthesia.

**Results:**

Righting and pedal withdrawal reflexes were lost within 3 and 5 min, respectively. Doses of sufentanil and midazolam were 0.48 μg/kg BW and 0.09 mg/kg BW for induction, and 0.72 μg/kg BW/h and 0.13 mg/kg BW/h for maintenance. Apnoea occurred in two rabbits. Induction of anaesthesia caused a significant increase in heart rate, cardiac output and arterial CO_2_ partial pressure and a decrease in mean arterial pressure, respiratory rate and pH. Mean time from stopping the infusion to endotracheal extubation was 5 min, and to return of the righting reflex 7 min. Anaesthesia was characterized by induction and recovery without excitation, with muscle relaxation, and absence of the pedal withdrawal reflex.

**Conclusions:**

TIVA with sufentanil-midazolam provided smooth induction and recovery of anaesthesia in rabbits but with marked hypotension and respiratory depression, requiring mechanical ventilation. Further evaluation is needed to establish if the protocol is useful for rabbits undergoing surgery.

## Background

The rabbit is the third most commonly used species for experimental research in the European Union, and it is increasing in numbers [1]. It is also the third most common pet species anaesthetised in the United Kingdom [2]. Rabbits are high risk anaesthesia patients, with a mortality risk 14 times higher than in dogs [3]. Possible reasons for this are that rabbits are easily stressed prey animals, they are difficult to intubate endotracheally and react to mask induction with volatile anaesthetics by extended breath-holding [4-6]. Rabbits have a large abdominal cavity in relation to the thoracic cavity, with the results that the pressure from the intestinal mass may interfere with respiration in dorsal recumbency during anaesthesia. Furthermore, obesity is increasing in pet rabbits, which also adds to the problem. Respiratory infection with *Pasteurella pneumotropica*, which is common in pet rabbits, may cause a reduction of the hydrogen ion driven respiratory drive, adding to the anaesthetic risk [7]. Rabbits are also prone to develop substantial hypotension during anaesthesia [8].

Anaesthesia of long-duration is often needed in experimental settings, as well as in clinical settings, as rabbit owners request more elaborate treatment for their pets. Inhalation anaesthesia is an option for long-duration anaesthesia although not all clinics or research institutions have access to the necessary facilities and equipment. Although significant research has been done to improve safety and reduce negative environmental impact, volatile anaesthetics may constitute an occupational health hazard and are toxic to the environment. Elaborate equipment is needed for waste gas scavenging, and there have been plans to phase out the use of volatile anaesthetics [9].

In recent years continuous total intravenous anaesthesia (TIVA) has become popular in veterinary anaesthesia [10]. Advantages compared to volatile anaesthesia are: reduced cardiovascular depression, smooth recovery and reduced incidence of postoperative nausea [11-13]. The development of intravenous (IV) anaesthetics with a short half-life has made it possible to control duration and depth of anaesthesia in a manner similar to inhalation anaesthesia.

Several TIVA protocols have been evaluated in animals, including the use of opioids, benzodiazepines, ketamine and propofol. Of the few that have been described in rabbits, TIVA with either ketamine-xylazine [14] or propofol [15,16] does not provide a surgical plane of anaesthesia, and yet produces substantial negative effects on cardiovascular variables. TIVA with fentanyl in combination with either diazepam [17] or propofol [18] has only been examined in combination with neuro-muscular blocking agents, which may prevent evaluation of analgesic properties. Ketamine-fentanyl TIVA has been used in combination with local anaesthesia in rabbits for cranial surgery [19], but the quality of anaesthesia was not reported.

High-dose opioid anaesthesia has been employed in experimental primate [20] and swine [21] surgery, with limited negative cardiovascular effects. Opioids inhibit sympathetic activity [22] and provide haemodynamic stability both during and after surgery. Sufentanil, which has been shown to inhibit sympathetic activity better than fentanyl in dogs [23], was used successfully in combination with the benzodiazepine midazolam for major surgery in dogs that were cardiovascularly compromised, and was shown to provide good hemodynamic stability [24]. One benefit of using TIVA with sufentanil-midazolam is that its effects may be antagonized with commercially available antidotes.

For the above-mentioned reasons, sufentanil-midazolam TIVA may be useful in rabbit anaesthesia. Our aim was to evaluate the cardiorespiratory effects of a TIVA protocol with sufentanil-midazolam in medetomidine premedicated NZW rabbits. Variables of anaesthesia induction, maintenance and recovery are reported together with cardio-respiratory variables and concentrations of serum glucose, lactate, total protein, sufentanil and midazolam.

## Results

Data are presented as mean ± SD or median (range) and n = 8 unless indicated. Haematology results are presented in Table 1.

**Table 1:** Haematology in eight New Zealand White Rabbits, sampled during physical restraint before anaesthesia

**Variable**^**a**^	**Mean ± SD**
EPC (10^12^/L)	4.7 ± 0.4
Hb (g/L)	102 ± 9
EVF (%)	27 ± 1
MCV (fL)	62 ± 3
MCHC (g/L)	350 ± 5
Reticulocytes (%)	2.0 ± 0.6
LPK (10^9^/L)	4.7 ± 0.8
Heterophils (10^9^/L)	1.5 ± 0.4
Eosinophils (10^9^/L)	0.2 ± 0.1
Basophils (10^9^/L)	0.4 ± 0.2
Lymphocytes (10^9^/L)	2.6 ± 0.7
Monocytes (10^9^/L)	0.1 ± 0.1

^a^*EPC* erythrocyte particle concentration, *Hb* haemoglobin, *LPK* leucocyte particle concentration, *EVF* erythrocyte volume fraction, *MCV* mean corpuscular volume, *MCHC* mean corpuscular hemoglobin concentration.

The righting reflex was lost in 3 ± 1 min and the pedal withdrawal reflex in 5 ± 2 min after start of induction. Induction occurred without excitation or apnoea, and the number of intubation attempts ranged between 2 and 6. No apnoea or struggling was encountered during intubation. Time to intubation from start of induction was 9 ± 2 min (n = 7). Intubation failed in one rabbit and a close fitting mask (Galemed with connector, Kruuse svenska AB) was used instead. Induction doses for sufentanil and midazolam were 0.48 ± 0.11 μg/kg BW and 0.09 ± 0.02 mg/kg BW, respectively. Maintenance doses were 0.72 ± 0.15 μg/kg BW/h and 0.13 ± 0.03 mg/kg BW/h respectively, corresponding to a mean infusion rate of 0.29 ± 0.06 ml/kg BW/h. The infusion rate did not change statistically over 60 min of anaesthesia duration.

**Figure 1:**
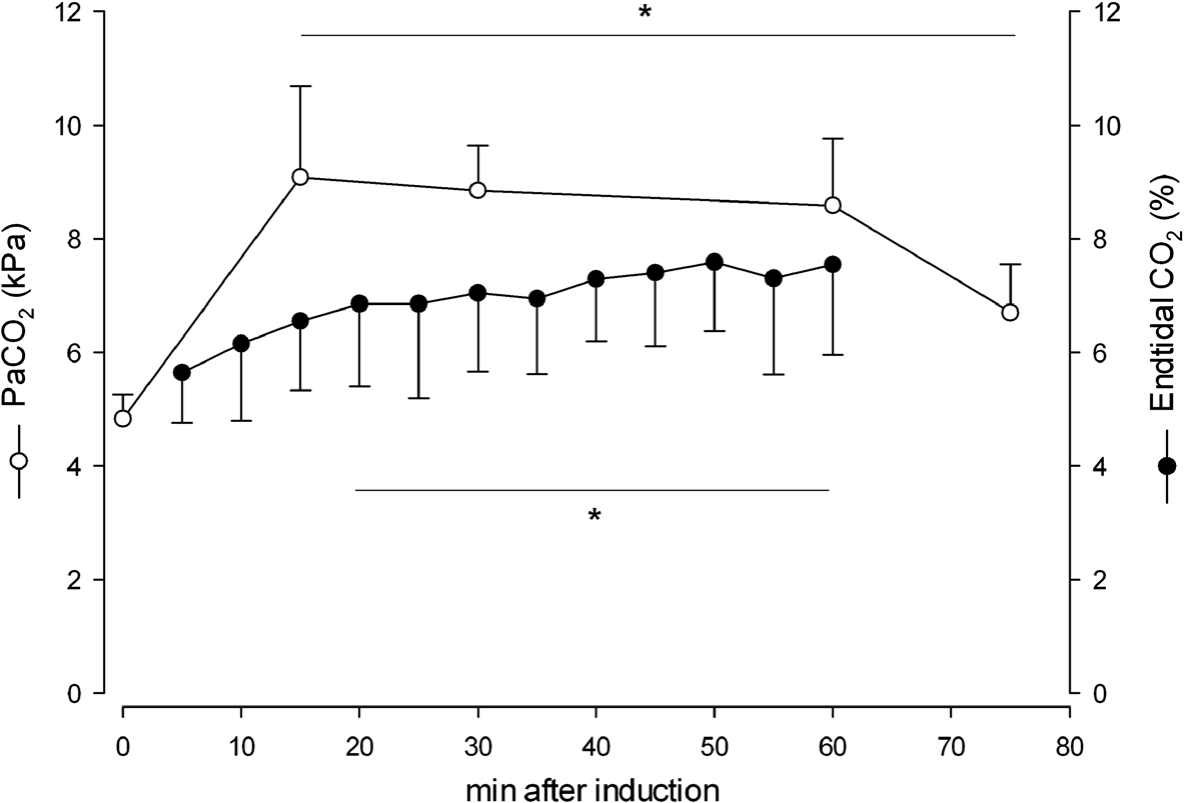
**Endtidal CO**_**2**_** and PaCO**_**2**_** (mean ± SD) in eight NZW rabbits during TIVA with sufentanil-midazolam.** *P < 0.05 compared with values recorded in sedated rabbits at 0 min (PaCO_2_) or 5 min after induction (ETCO_2_). One Way Repeated Measures Anova, multiple comparisons with Holm-Sidak’s Method.

**Figure 2:**
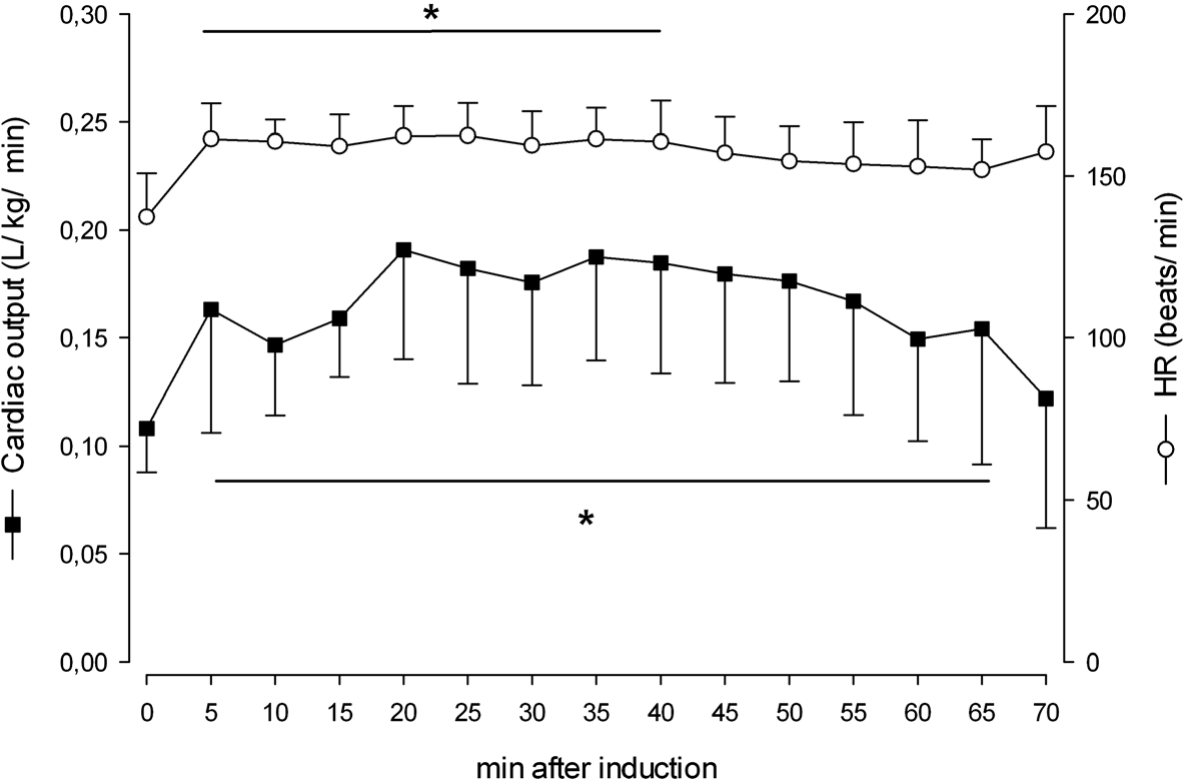
**Cardiac output (n = 6) and heart rate (n = 8) in medetomidine premedicated NZW rabbits during TIVA with sufentanil-midazolam (mean ± SD).** *p < 0.05 compared with values recorded in sedated rabbits at 0 min. One-way ANOVA for repeated measures, posthoc test Holm-Sidak.

Maintenance of anaesthesia was characterized by muscle relaxation and loss of the pedal withdrawal reflex. Compared to values during sedation, the respiratory rate was reduced during the first 20 minutes of anaesthesia (Table 2). Two rabbits occasionally needed mechanical ventilation during maintenance due to apnoea. End-tidal CO_2_ (ETCO_2_) and arterial partial pressure of CO_2_ (PaCO_2_) were increased during most of the maintenance of anaesthesia (Figure 1). Blood pH decreased from 7.50 ± 0.00 to 7.28 ± 0.04 and serum lactate from 1.9 ± 0.6 to 0.7 ± 0.2 mmol/L (n = 7). The arterial partial pressure of O_2_ (PaO_2_) increased from 9.6 (8.4-11.1) to 38 (35–46) kPa whereas mean arterial pressure (MAP) decreased during anaesthesia (Table 2). Heart rate (HR) and cardiac output (CO, n = 6) increased immediately after induction and remained high during most of maintenance (Figure 2). No signs of arrhythmias were observed on the ECG.

**Table 2:** Respiratory rate and mean arterial pressure recorded before, during and after sufentanil-midazolam TIVA in medetomidine-premedicated New Zealand White rabbits (n = 8)

**Time point**	**Respiratory rate (per min)**	**Mean arterial pressure (mmHg)**
sedated^a^	100 (40–240)	70 (61–78)
5	4^b^ (0–22)	56 (49–73)
10	6^b^ (1–26)	55 (48–63)
20	6^b^ (0–22)	50^b^ (41–60)
30	12 (6–17)	49 (42–57)
40	13 (8–24)	46^b^ (39–54)
50	10^b^ (0–14)	48^b^ (37–53)
60	13 (1–24)	49^b^ (40–60)
70*	24 (12–130)	57 (44–72)

Values are presented as median (range).

^a^values were recorded approximately 30 min after injection with medetomidine (0.1 mg/kg BW SC). ^b^significantly different compared with values recorded in sedated rabbits. Friedman Repeated Measures ANOVA on ranks, multiple comparisons with Dunn’s method. *10 min after TIVA stop.

**Figure 3:**
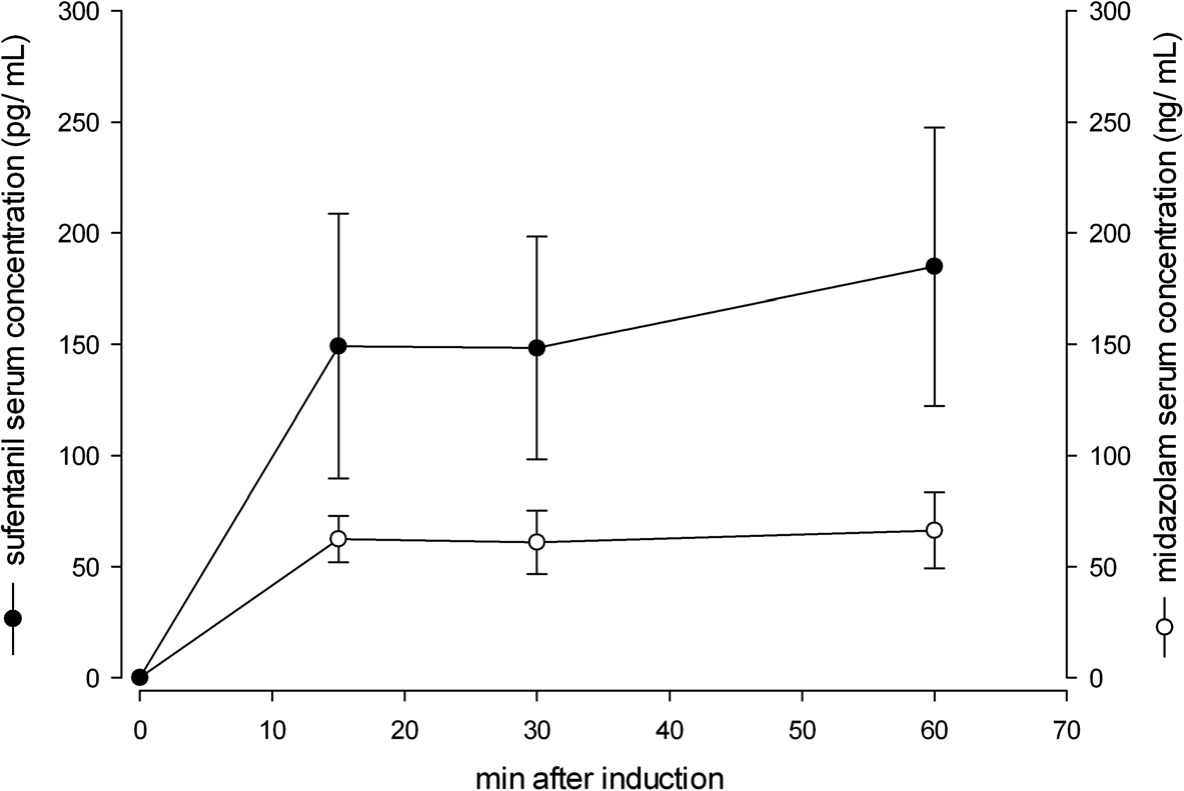
Serum levels of sufentanil and midazolam during TIVA in medetomidine premedicated NZW rabbits (n = 8).

Rectal temperature dropped from 39.2°C (37.9–39.9) to 38.4°C (38.1–39.0) at 60 min. Blood glucose increased from 10.8 (6.6–14.0) to 16.0 (8.3-18.5) mmol/L (n = 7) and haematocrit from 23 ± 2 to 27 ± 1%. Sufentanil and midazolam serum levels did not change from 15 to 60 min during anaesthesia (Figure 3). Time from stop of TIVA infusion to extubation was 5 ± 3 min, and to recovery of the righting reflex 7 ± 6 min. Recovery was without excitation. Body weight was reduced by 3% 24 h after anaesthesia and restored 48 h later. No clinical abnormalities were seen during the week after anaesthesia.

## Discussion

TIVA with sufentanil-midazolam was evaluated in medetomidine pre-medicated NZW rabbits. Induction was without excitation, apnoea or muscle rigidity, providing good conditions for endotracheal intubation. Anaesthesia maintenance was characterised by absence of a pedal withdrawal reflex and muscle relaxation, although marked respiratory depression and hypotension were produced.

We successfully intubated the airways of 6 rabbits after 2–4 blind attempts. The conditions for intubation were good, as judged by lack of jaw muscle tone and reflex responses to intubation, and when an intubation attempt failed, the endotracheal tube ended up in the oesophagus. No laryngospasm, swelling of the larynx or bleeding was encountered. The difficulty of endotracheal intubation in rabbits has been described in numerous studies [25-27]. Rabbits have a narrow oral cavity which makes visual intubation difficult and successful blind intubation requires both practice and routine. Using a specially designed laryngoscope such as the Flecknell™ small animal laryngoscope (Alstoe Ltd. Animal Health, UK) may be helpful.

Apnoea during induction must be avoided, since it complicates blind intubation and rapidly leads to hypoxemia in rabbits, due to their high metabolic rate. To avoid apnoea, anaesthesia must be induced slowly. Midazolam alone can cause apnoea in humans if infused too quickly IV [28]. Slow induction also prevents muscle rigidity which occurs if sufentanil is administered too fast, which has been shown in pigs [21] and humans. In the currents study, catalepsy was not observed.

A similar IV drug combination (xylazine-alfentanyl-midazolam) has been evaluated in NZW rabbits [29], although not as a continuous infusion. The effects on the respiratory and cardiovascular systems were similar to those seen in the present study, but muscle relaxation was poor and seizures were induced. In comparison, TIVA with sufentanil-midazolam seems to be a better alternative.

During development of the current protocol, premedication with fentanyl-fluanisone was abandoned due to occurrence of apnoea during induction of anaesthesia. This was not the case after premedication with medetomidine at 0.1 mg/kg BW. In combination with local anaesthesia cream, medetomidine sedation produced satisfactory conditions for placement of vessel catheters. During TIVA maintenance however, apnoea occurred occasionally in two rabbits.

Medetomidine is known for its adverse cardiovascular effects [30], even in very low doses [31]. In human anaesthesiology, alpha-2-adrenergic agonists are increasingly used in low doses to improve cardiovascular stability and prevent tachycardia [32].

Sufentanil-midazolam anaesthesia caused a marked decrease in respiratory rate and marked hypercapnia. The level of hypercapnia produced was similar to that produced by 2 MAC isoflurane [33]. Opioids are known respiratory depressants, and midazolam has been shown to slightly add to this depression in rabbits [34]. Despite marked respiratory depression, we chose to ventilate the rabbits minimally in order to evaluate the extent of respiratory depression. It is likely that the respiratory depression had an effect on other variables, such as the HR. However, experience shows that during short term anaesthesia, some degree of hypercapnia (6–7 kPa) is well tolerated in healthy cats and dogs [35], and may even be beneficial since it stimulates respiration and increases blood flow to the brain [36]. The occurrence of apnoea during maintenance of anaesthesia necessitates the use of mechanical ventilation, which is the main disadvantage of the protocol since it requires successful endotracheal intubation as well as the knowledge and skills of using a ventilator.

The heart rate increased after induction with TIVA, although the values did not reach the levels reported in awake unrestrained rabbits [37]. A possible explanation for the increase is the sympathomimetic stimulation caused by endotracheal intubation [38]. Alpha-2-agonists are able to block cardiovascular responses caused by intubation and surgery in humans [39] and opioids can block the sympathetic response to surgery in dogs [23,24]. If intubation caused the increased HR in the present study, the medetomidine and sufentanil doses used must have been insufficient to prevent the response. The simultaneous hypercapnia and hypotension that developed may also have contributed to the increase in HR. Isoflurane increases heart rate by 20% because of depression of the vagal tone rather than an increase in the sympathetic tone [40].

Rather than an increase in HR we had anticipated a decrease, since opioids are known to cause bradycardia through medullary vagal stimulation [41]. Sufentanil-medetomidine anaesthesia, albeit in a higher doses than in the present study and without the concurrent use of an alpha-2-adrenergic agonist, causes bradycardia in dogs [24] as well as humans [42].

Marked hypotension developed during anaesthesia (MAP 46 mm Hg), yet all rabbits recovered uneventfully. A high prevalence of hypotension, defined as a MAP < 60 mm Hg, has been reported in rabbits anaesthetized with e.g. isoflurane, haltothane or ketamine-medetomidine [8,33,43]. Maintenance of anaesthesia with isoflurane in NZW rabbits will give rise to MAP in the range of 33–82 mmHg [8]. The pressure measured in the auricular artery has been shown to be approximately 8 mm Hg lower than in the carotid artery [38]. In the present study MAP in the sedated rabbits (~70 mmHg) was lower than reported in non-sedated unrestrained resting rabbits (~80 mmHg), [37] and similar to MAP in rabbits after IV administration of 10 μg/kg BW dexmedetomidine (~75 mmHg), [44]. Midazolam alone [45] or in combination with sufentanil [24], has been shown to decrease vascular resistance and blood pressure in dogs, however MAP did not fall below 65 mmHg [24]. In humans, sufentanil-midazolam also decreases MAP (~75 mm Hg), [46,47]. In contrast to the present study, the human and dog studies included major surgery, which may have affected MAP. Uncorrected hypotension may contribute to the high mortality levels in rabbit anaesthesia [8] and cardiovascular support, which is routine in other animal species during anaesthesia, may possibly decrease mortality rates.

MAP and measurement of lactate concentrations [48] are indirect measures of tissue perfusion. The serum lactate levels in the present study indicate that tissue perfusion was adequate despite the low MAP. The levels were low before and during anaesthesia (0.7-1.9 mmol/L), similar to those seen in rabbits lightly anaesthetised with propofol (1.3 mmol/L), [49] and considerably lower compared to non-sedated, restrained rabbits (7.3 mmol/L), [50]. Increased lactate levels are a sign of tissue hypoxia and anaerobic metabolism and should be treated with therapy directed at improving tissue oxygen delivery. The acidosis that quickly developed after induction of anaesthesia was most likely of respiratory origin.

Tissue perfusion is likely to be compromised if vasoconstriction and reduced cardiac output are present. In the current study, however, CO increased after anaesthesia induction, which was likely a consequence of the increased HR and decreased MAP. Midazolam has been shown to increase cardiac output in dogs at doses as low as 1 mg/kg [45].

CO was measured with the lithium-dilution technique, which has not yet been validated in rabbits. It has been validated in human babies with a similar BW (~2 kg) and found to be accurate in comparison to transpulmonary thermodilution [51]. It has also been validated in dogs [52] and cats [53]. The benefit of the technique is that a peripheral instead of a central vein may be used, which reduces the risk for complications. No adverse effects were seen due to the CO measurement in the present study. Values recorded in medetomidine sedated rabbits were similar to levels measured with a transit time Doppler in NZW rabbits sedated with dexmedetomidine 0.01 mg/kg BWIV [44].

The glucose levels in pre-medicated rabbits were high (7–14 mmol/L) compared to those reported in non-sedated rabbits (4–8 mmol/L), [54]. A further increase in glucose was seen after anaesthesia induction, reaching a maximum of 8–18 mmol/L at approximately 50 min after medetomidine administration. Medetomidine is known to decrease insulin levels [55] and has been shown to increase blood glucose levels in rabbits [56]. Midazolam on the other hand, has been shown to decrease glucose levels during surgery in humans [57] and, furthermore opioids inhibit the entire sympathetic nervous system [22]. If the sufentanil-midazolam doses were not high enough to fully inhibit sympathetic activity, the stimulation caused by endotracheal intubation may have contributed to the increase in glucose levels.

Serum concentrations of sufentanil (0.60 ng/mL) and midazolam (140 ng/mL) remained constant throughout anaesthesia, with no tendency to accumulate. Plasma concentrations have been measured in humans undergoing heart surgery during sufentanil-midazolam anaesthesia [46]. Compared to the present study, the infusion level was higher for sufentanil and lower for midazolam and accordingly, the plasma levels higher for sufentanil (2–4 ng/mL) and lower for midazolam (75–100 ng/mL).

To further evaluate this protocol, experimental work needs to be undertaken in both healthy and sick rabbits during surgery and in comparison with inhalation anaesthesia.

## Conclusions

TIVA with sufentanil-midazolam provided smooth induction and recovery of anaesthesia in rabbits, but with marked hypotension and respiratory depression, requiring mechanical ventilation. Despite the side effects, the protocol may still be an alternative to inhalation anaesthesia in situations where longer duration anaesthesia is needed and inhalation anaesthesia is not an option. The fact that endotracheal intubation and mechanical ventilation is necessary makes the protocol a less likely alternative for the general practitioner.

## Methods

The study was approved by Uppsala, Sweden, Experimental Animal Ethics Committee (C368/9).

### Animals

Nine female New Zealand White (NZW) rabbits (HB Lidköpings kaninfarm, Lidköping, Sweden), aged 6.9 ± 0.6 months and with a BW of 3.9 ± 0.3 kg, were acclimatised to the user animal facility for 3 weeks and accustomed to handling. The body weight was recorded daily for five days before the study. The breeder’s colony was health- monitored according to European guidelines [58] and was free from common rabbit pathogens (rabbit hemorrhagic disease virus, rabbit rotavirus/ rabbit coronavirus, *B*. *bronchiseptica*, *C*. *piliforme*, *P*. *multocida*, *Salmonella spp*, endo- and ectoparasites, dermatophytes). Clinical examination and complete blood cell count (Table 1) on EDTA-blood with ADVIA®Automated Solutions (Siemens Healthcare Diagnostics) were performed before anaesthesia to exclude subclinical disease. White blood cell differential counts were also performed manually for quality control.

Due to an unequal number, all but one rabbit were pair-housed in floor-pens of 3.2 m^2^ with autoclaved wood shavings and a shelter for hiding. Pens were cleaned weekly. Each rabbit was fed 200 mL of pellets daily (Lactamin K1, Lactamin AB). Autoclaved hay and water were provided *ad lib*. The light–dark-cycle was 12:12 h with lights on at 0600 h. Room temperature was 20 ± 2°C and humidity 55 ± 10%.

### Development of the protocol

In a pilot study, the rabbits were anaesthetized once with dose combinations which were gradually adjusted between individuals according to observed reactions. The doses were extrapolated from studies of sufentanil and/or midazolam in rabbits or dogs [24,59,60]. Pre-medication with 0.1-0.2 mL fentanyl-fluanisone/kg BW (Hypnorm, Jansen Cilag, UK, n = 3) was associated with apnoea during TIVA induction and was therefore replaced with 0.1 mg medetomidine/kg BW (Domitor, 1 mg/mL, Orion Corporation, Finland, n = 6), administered subcutaneously (SC). Anaesthesia was induced IV with different concentrations of sufentanil (5 and 50 μg/mL, Jansen-Cilag) and midazolam (1 and 5 mg/mL, Actavis AB) in physiological saline (1 rabbit/dose). Four different sufentanil-midazolam dose combinations were evaluated and based on the degree of respiratory depression and muscle rigidity, the most promising combination was further evaluated in the main study, after a wash-out period of at least 12 days. One rabbit premedicated with fluanisone-fentanyl died from apnoea during TIVA induction before any rescue attempts were initiated, thus leaving 8 rabbits for the main study.

### Instrumentation and anaesthesia

Local anaesthetic cream was applied on both ears 45 min before catheterization of the ear vein and artery. The rabbits were premedicated SC with medetomidine (0.1 mg/kg BW) 30 min before catheterisation. Premedication was administered while the rabbits were in the animal holding room.

In the sedated rabbit, one arterial (Insyte-W catheter, 22 G, BD AB) and two venous (Neoflon 24 G, BD AB) catheters were placed in the ears after removal of the fur. The intravenous catheters were used for anaesthesia infusion and fluid replacement, and the arterial catheter was used for monitoring of blood pressure and collection of arterial blood. Self-adhesive ECG electrodes (GM Health Care) were placed laterally on the elbows and the left knee and a human pulse oximeter finger clip sensor (Datex-Ohmeda) on the tail after clipping of fur. A temperature probe was placed in the rectum and connected to the Datex system.

Anaesthesia was induced with a mixture of sufentanil and midazolam. For this, 1 mL of sufentanil (50 μg/mL) and 10 mL of midazolam (1 mg/mL) was mixed with 11 mL of physiological saline, resulting in a concentration of 2.3 μg/mL sufentanil and 0.45 mg/mL midazolam. Infusion was started at a flow rate of 0.3 mL/kg BW/h with a syringe pump (Braun Compact S). After 1 minute, 0.1 mL boluses of the same mixture were administered IV every 20 seconds, until induction was complete, as judged by loss of the righting reflex and muscle relaxation (loss of jaw and limb muscle tone). During induction, the rabbit was breathing 100% oxygen (2 L/min) from a face mask. Starting the infusion before bolus administration has been shown to prevent muscle rigidity, which is commonly caused by opioids [21].

The larynx was then sprayed with lidocain (4–8 mg, Intubeaze, Dechra Veterinary Products) and the airways intubated blindly with an uncuffed endotracheal tube (outer diameter 3.3–4.0 mm, Kruuse Svenska AB). One intubation attempt was defined as insertion of the endotracheal tube in its full length. A tube providing oxygen (2 L/min) was held in front of the rabbit’s nares during intubation. The tube was connected to the anaesthesia machine (Anmedic Q-Circle System) via a paediatric circular breathing system (Intersurgical Ltd) and the rabbit was placed in dorsal recumbency. The rabbit was left to breathe spontaneously, receiving 67% oxygen in air at a flow rate of 3 L/min. In case of apnoea (30 s without a breath), a reservoir bag (1 L) was carefully compressed every 30 s, until spontaneous breathing resumed. The time from start of infusion to loss of righting and pedal reflexes were recorded, as well as the number of successful intubations and intubation attempts.

Anaesthesia was maintained for 60 min. Every 5 min the infusion rate of the TIVA solution was adjusted according to nociceptive response (pinching of ear and skin between toes on hind foot). In case of a positive pedal withdrawal reflex or voluntary movement, the infusion rate was increased by 10%. If respiratory rate alone increased, the flow rate was left unchanged. If there was no reaction, the infusion rate was decreased by 10%. To minimize temperature loss during anaesthesia the rabbits were placed on a heating blanket. Warmed Ringer acetate solution (Fresenius Kabi, Sweden) was administered IV at a rate of 20 mL/kg BW/h.

To end anaesthesia, the sufentanil-midazolam infusion was discontinued and times to extubation and return of the righting reflex were recorded. The rabbits recovered under a heat lamp (24–26°C) before being returned to their home pens. During the week following anaesthesia, the rabbits were examined and weighed daily.

### Measurements of haemodynamic and respiratory variables

Systemic blood pressure was recorded by connecting the arterial catheter to a pressure transducer (Gabartith, BD), which was calibrated to air at the level of the heart. Together with the ECG and pulse oximeter, it was connected to a monitor (CS/3, Datex-Engstrom) for continuous recording and storage on a PC (software: Datex-Ohmeda S/5 Collect, GE Healthcare Sverige AB). Heart rate (HR) was recorded from the ECG or pulse oximeter during anaesthesia.

Monitoring of cardiac output (CO) was achieved by pulse contour analysis using an indicator dilution technique (LiDCO System, LiDCO ltd). To calibrate the system, a bolus dose of 5.6 μmol/kg BW lithium chloride (0.15 mmol/mL) was injected IV through the ear vein catheter. A lithium dilution curve was obtained by withdrawing blood past the lithium-sensor attached to the existing arterial line. Using the pulse contour analysis software (PulseCO™system, incorporated in the LiDCO™plus Monitor), CO was continuously displayed on the monitor and recorded. End tidal CO_2_ (ETCO_2_) was monitored using sidestream capnometry (Datex, CS/3 monitor). Respiratory rate was obtained from the capnometry readings of the Datex monitor.

### Sampling and analyses of blood samples

Arterial blood samples were used for measurement of haematology, blood gases, pH, lactate, glucose, drug concentrations and total protein at predefined time points before, during and after anaesthesia. In total ≤ 7.5 mL of blood was collected per kg BW. A portable analyser (CG8 + cartridges, i-STAT®1 Portable Clinical Analyzer, Abbott Laboratories) was used for immediate analysis of haematocrit, pH, partial pressure of arterial CO_2_ (PaCO_2_) and O_2_ (PaO_2_), in blood (0.1 mL) collected anaerobically into non-heparinised syringes. Blood gas and pH values were corrected to rectal temperature.

Serum lactate concentrations were measured using Analox lysing tubes and an Analox Instruments GM7 analyzer. Serum was harvested after centrifugation of blood for 10 min at 3000 rpm (Universal 16R, Hettich Labinstrument) and stored at −80°C until analysis. Sufentanil and midazolam serum concentrations were measured using reversed-phase and hydrophilic interaction chromatography and tandem mass spectrometry (ACQUITY UPLC® BEH Shield RP18 and UPLC/ MS MS, Waters Sverige AB). Total serum protein was measured by refractometry (Master, ATAGO, Kruuse, Sweden).

### Sampling procedure

Before induction of anaesthesia, in the sedated rabbit respiratory rate (RR), heart rate (HR), mean arterial pressure (MAP), CO and pulse oximetry derived haemoglobin oxygen saturation (SpO_2_) were measured. Arterial blood was sampled for analyses of blood gases, lactate, total protein and glucose. During anaesthesia RR, HR, MAP, CO, SpO_2,_ ETCO_2_ and BT were recorded every 10 minutes. Arterial blood samples were taken for analysis of blood gases, lactate, drug concentrations, total protein and glucose every 15 minutes during anaesthesia and 15 min after discontinuation of the TIVA-infusion.

### Statistical analyses

Analysis (Sigma Plot, Systat Software Inc.) over time was performed with one-way ANOVA for repeated measures (continuous data, normally distributed by test of Shapiro-Wilk) or Friedman repeated measures ANOVA on ranks (discontinuous data or non-normally distributed data) with Holm-Sidak’s or Dunn’s posthoc tests, respectively (multiple comparisons versus pre-induction). Pre-induction values were recorded 30 min after SC injection with medetomidine. For ETCO_2_, the first value used for the comparison over time was recorded 5 min after induction. A P-value < 0.05 was considered significant. Continuous and normally distributed data are presented as mean ± SD and discontinuous or non-normal distributed data as median (range).

## Competing interests

The authors declare that they have no competing interests.

## Authors’ contributions

AE, PH and MJW designed the study and all authors contributed to the acquisition of data. PH analysed the data and all authors contributed to interpretation of data, drafting and revision of the manuscript. All authors read and approved the final manuscript.
